# Migration for obstetric care: the impact of regional Obstetric Care Facility Density disparities in Tanzania

**DOI:** 10.1186/s13104-018-3780-0

**Published:** 2018-09-21

**Authors:** Projestine Selestine Muganyizi, Edward Maswanya, Stella Kilima, Ahmad Makuwani

**Affiliations:** 1 Department of Obstetrics and Gynecology, Muhimbili University of Health and Allied Sciences (MUHAS), Dar es Salaam, Tanzania; 2 National Medical Research Institute, Dar es Salaam, Tanzania; 3 Ministry of Health Community Development Gender Elderly and Children, Dar es Salaam, Tanzania

**Keywords:** Obstetric care services, Facility density, Institutional delivery, Migration, Tanzania

## Abstract

**Objective:**

This is an extended analysis of the previously published data to demonstrate the relationship between high Obstetric Care Facility Density (OCFD) and migration for obstetric services in Tanzania.

**Results:**

Overall, regions with excess institutional deliveries had significantly higher OCFD compared to other regions. A consistent pattern was observed whereby regions with excess Institutional deliveries also exhibited the most outstanding OCFD of all the neighbouring regions. The observed patterns of Institutional deliveries and OCFD affirm the hypothesis of immigration for obstetric care services from low to high OCFD regions. Further research is suggested to prove this hypothesis in the field.

## Introduction

For the purpose of this analysis, Obstetric Care Facility Density (OCFD) is defined as the number of health facilities that provide obstetric care services per 500,000 population. This definition has been derived from the United Nations (UN) process indicators for availability and geographical distribution of Basic and Comprehensive Emergency Obstetric and Neonatal Care (EmONC) facilities per 500,000 population based on National or Sub-National’s most recent population data [1, 2]. The UN process indicators have been blamed for inability to capture spatial aspects of EmONC service availability and failure in capturing an important role played by other obstetric care facilities in an area not qualifying as EmONC [3]. Although OCFD is not one of the UN process indicators, it is a proxy to spatial distribution of obstetric care services and encompasses the contribution of all obstetric care facilities in a geographical area regardless of their EmONC statuses [1, 3, 4]. In a National Survey of EmONC indicators in Tanzania in 2015, overall OCFD was shown to be strongly correlated with institutional delivery [4]. Moreover, among the 25 regions, four had institutional deliveries in excess of 100% of the expected. These regions were Mara, Lindi, Pwani and Njombe [5]. Since the estimation of institutional deliveries was calculated based on regional population and its crude birth rate [6], deliveries in excess of 100% could be a reflection of migration of women from the low OCFD neighbourhood to seek obstetric care services in areas with high OCFD. This phenomenon of migration from low OCFD settings to high OCFD to seek care has not been demonstrated in literature. This article extends our previous work in Tanzania by further exploring the relationship between OCFD and excessive institutional deliveries.

## Main text

### Methods

Institutional delivery rate was estimated as a percentage of all registered births in the region that took place in all the obstetric care facilities. In the survey, the numerator was obtained by recording all births that took place in obstetric care facilities during the past 1 year. The denominator was calculated for each region from the total population with its Crude birth rate, both obtained from Tanzania Bureau of Statistics based on the 2012 Population and Housing Census. The survey was conducted in all obstetric care facilities in Tanzania Mainland in 2015 whose details are given in the previous publications [4, 5]. Obstetric care facilities included dispensaries, health centres and hospitals responsible for provision of maternity care and delivery services.

The aim of this extended analysis is to demonstrate statistically if the overall distribution of OCFD differs between regions with institutional deliveries equal or less than 100% and those with institutional deliveries greater than 100% (henceforth referred as to excessive deliveries). A two sided Mann–Whitney U test for independent samples is used to test the hypothesis. Further, mapping of institutional deliveries in the neighbourhood regions with excessive deliveries was done to help describe the connotation that regions with the highest OCFD in a zone attracts more deliveries from the neighbourhood leading to overcrowding of obstetric care facilities. The implications of Geographical inequity of OCFD on the burden to obstetric care services in high OCFD regions are discussed.

### Results and discussion

The Mann–Whitney U test (Table 1) rejects the null hypothesis that the distribution of OCFD among regions with excessive deliveries and those with the expected normal burden of 100% or less is the same. Regions with more than 100% institutional deliveries were then located in a geographical map and OCFDs of the neighbouring regions studied (attached as extra files). The distribution of institutional deliveries and OCFD in the neighbourhood regions with excessive institutional deliveries is demonstrated in Tables 2 and 3.

**Table 1:** Comparison of population obstetric care facility densities among regions with excess and normal institutional deliveries (ID) in Tanzania

Region	Normal, ID ≤ 100% (n = 21) Rank	Obstetric care facilities per 500,000 population for ID ≤ 100%	Obstetric care facilities per 500,000 population for ID > 100%	Excess, ID > 100% (n = 4) Rank
Dar es Salaam	1	16		
Geita	2	33		
Kigoma	3	37		
Mwanza	4	44		
Simiyu	5	48		
Manyara	6	51		
Shinyanga	7	52		
Kagera	8	53		
Katavi	9	55		
Tabora	10	56		
Arusha	11	58		
Iringa	12	60		
Morogoro	13	63		
Mbeya	14	69		
Singida	15	71		
Dodoma	16	73		
Ruvuma	17.5	74		
Tanga	17.5	74		
Mtwara	19	75		
Kilimanjaro	20	76		
Mara			77	21
Rukwa	22	95		
Pwani			109	23
Lindi			121	24
Njombe			150	25
	Sum = 232			Sum = 93

**Table 2:** Rates of institutional deliveries in the neighbourhood of regions with excessive institutional deliveries in Tanzania

Regions with excessive institutional deliveries	OCFD in closest neighbourhood regions			
Lindi (102%)	Ruvuma (93%)	Mtwara (91%)		
Mara (107%)	Arusha (69%)	Simiyu (46%)	Kenya border	
Pwani (166%)	Dar es salaam (98%)	Tanga (66%)	Morogoro (84%)	
Njombe (110%)	Ruvuma (93%)	Iringa (77%)	Morogoro (84%)	Mbeya (93%)

**Table 3:** Distribution of OCFD in the neighbourhood of regions with excessive institution deliveries in Tanzania

Regions with highest OCFD per 500,000 population	Neighbourhood regions (OCFD)			
Lindi (121.4)	Ruvuma (74.1)	Mtwara (75.1)		
Mara (77.1)	Arusha (57.5)	Simiyu (47.7)	Kenya border	
Pwani (108.8)	Dar es salaam (15.5)	Tanga (74.3)	Morogoro (62.7)	
Njombe (149.6)	Ruvuma (74.1)	Iringa (59.5)	Morogoro (62.7)	Mbeya (69.4)

Independent samples Mann–Whitney U test, p = 0.000 thus rejecting the null hypothesis that the distribution of density is the same across regions with ID ≤ 100% and ID > 100%.

Regions with excessive institutional deliveries (Table 2) also exhibited the most outstanding OCFD in their locations (Table 3) which supports a high correlation of the two. This affirms the hypothesis that migration for obstetric care services from low OCFD to high OCFD regions could be an important driver for excessive institutional deliveries in high OCFD regions. Since OCFD is a proxy for distance a high OCFD improves geographical access to services thus promoting utilisation of maternity services [7–10].

The regions with excessive institutional deliveries in Table 2 exhibit the most outstanding OCFD (Table 3) among the regions in their locations. Mara region was also neighbouring Tanzania-Kenyan border but the Institutional delivery rates, OCFD on the Kenyan side and the easiness to migrate across-border were not determined.

It is unlikely that OCFD is the only factor that accounts for excessive institutional deliveries in the four regions. The OCFD disparities seen among the regions with excessive institutional deliveries and their neighbourhoods could be a manifestation of economic inequity among them, which could also lead to better quality of obstetric care services in these 4 regions. However, in the primary analysis of the survey data, measures of EmONC quality of care such as availability of functional basic EmONC functions, Case fatality rate and caesarean section rates were not consistent with these four compared to the neighbourhood. This underscores the importance of OCFD as a cause for migration rather than the quality of service provided by the facilities in the four regions.

## Limitations and implications

One of the limitations of this analysis is the failure to establish the threshold of OCFD disparity level that triggers migration for obstetric care services from the neighbourhood. Moreover, the 2015 survey did not interview service providers and women seeking obstetric care services in order to demonstrate the existence of cross-border migration and the motives behind it. Nevertheless, the implication is that, inequitable distribution of OCFD among regions can ultimately lead to overcrowded maternity services and delivery rooms and overwhelmed resources, thus defeating the primary intention of improved quality of obstetric care and this contradicts with UN process indicators that advocates for 500,000 population to served with 4 Basic and 1 Comprehensive EmONC facilities. Future studies should include surveys of Obstetric care facilities to confirm the migration for obstetric care services, other factors that contribute to excessive institutional deliveries in the four regions and the economic impact of this migration if it real exists.

## Abbreviations

EmONC: Emergency Obstetric and Neonatal Care
ID: institutional deliveries
MOHCDGEC: Ministry of Health, Community Development, Gender, Elderly and Children
OCFD: Obstetric Care Facility Density
UN: United Nations
